# Synthesis of Hexagonal Structured GaS Nanosheets for Robust Femtosecond Pulse Generation

**DOI:** 10.3390/nano12030378

**Published:** 2022-01-24

**Authors:** Kun Guo, Qiang Yu, Fangqi Liu, Haiqin Deng, Tianan Yi, Bo Ren, Wei Su, Sicong Zhu, Zhiqiang Wang, Jian Wu, Pu Zhou

**Affiliations:** 1 College of Advanced Interdisciplinary Studies, National University of Defense Technology, Changsha 410073, China; guokun16@163.com (K.G.); qyu2015@sinano.ac.cn (Q.Y.); ddhq9859@163.com (H.D.); rbone725@163.com (B.R.); zhoupu203@163.com (P.Z.); 2 Hubei Province Key Laboratory of Systems Science in Metallurgical Process, College of Science, The State Key Laboratory for Refractories and Metallurgy, Wuhan University of Science and Technology, Wuhan 430081, China; lfq7@wust.edu.cn (F.L.); sczhu@wust.edu.cn (S.Z.); 3 College of Mechanical and Electrical Engineering, Hohai University, Changzhou 213022, China; 191319010004@hhu.edu.cn (T.Y.); opticsu@hhu.edu.cn (W.S.); 4 Aston Institute of Photonic Technologies, Aston University, Birmingham B4 7ET, UK; 5 Advanced Photonic Technology Lab, College of Electronic and Optical Engineering, Nanjing University of Posts and Telecommunications, Nanjing 210023, China

**Keywords:** Gallium sulfide, saturable absorber, mode-locked fiber laser, ultrafast pulse

## Abstract

Gallium sulfide (GaS), with a hexagonal structure, has received extensive attention due to its graphene-like structure and derived optical properties. Here, high-quality GaS was obtained via chemical vapor synthesis and then prepared as a saturable absorber by the stamp-assisted localization-transfer technique onto fiber end face. The stability of the material and the laser damage threshold are maintained due to the optimized thickness and the cavity integration form. The potential of the GaS for nonlinear optics is explored by constructing a GaS-based Erbium-doped mode-locked fiber laser. Stable femtosecond (~448 fs) mode-locking operation of the single pulse train is achieved, and the robust mode-locked operation (>30 days) was recorded. Experimental results show the potential of GaS for multi-functional ultrafast high-power lasers and promote continuous research on graphene-like materials in nonlinear optics and photonics.

## 1. Introduction

Ultrafast lasers that enable the generation of ultrashort energetic pulses have become indispensable tools for a variety of applications, such as industrial fabrication [1,2], biomedical diagnosis [3,4], controllable processing of materials [5,6], pulse measurement [7,8], gas-phase thermometry, and species concentration measurements [9]. One common method for producing ultrashort pulses in ultrafast lasers is the passive method that introduces a modulation of the intracavity field by placing a saturable absorber (SA) element in the cavity [10,11,12,13]. The SA can be classified as either “artificial” SA that uses a nonlinearity effect, such as the nonlinear Kerr effect, to create an intensity-dependent transmission, or real SA, that relies on the nonlinear absorption of materials [14]. Compared to ultrafast lasers with artificial SAs [15], ultrafast fiber lasers mode-locked by using real SA are of particular interest for practical applications in terms of the simple laser configuration as well as the polarization-independent operation and robustness. Although being mostly used for the generation of pulses by the passive mode-locking of lasers, semiconductor saturable absorber mirrors (SESAM) only work in a small wavelength range once fabricated [16,17,18]. One of the approaches for overcoming the narrow working wavelength range of SESAM is to employ lower-dimensional materials as SAs for the generating of ultrashort pulses, as the low-dimensional materials usually have ultrabroad spectral features of absorption. Furthermore, the properties of material-based SA such as the linear loss, the modulation depth, and the working wavelength can also be optimized in the fabrication process [19]. To date, graphene [20,21], topological insulator (TI) [22,23,24], transition metal dichalcogenides (TMDCs) [25,26,27], Mxene [28,29], bismuthine [30,31,32], and black phosphorus (BP) [33] have been applied in ultrafast fiber lasers [34,35,36,37]. Previous studies mostly focus on how the pulse width can be greatly compressed, for example, adding positive dispersion fiber for dispersion management [38,39]. It is worth mentioning that the robust factor in the pulsed laser needs further discussion, which will directly affect the subsequent application explore. The performance boost of ultrafast lasers is usually along the proposal of new mode-locking mechanisms as well as the discovery of the new materials for mode-locking operation.

Following the demand for stability and robustness in practical applications, real SA choosing is important [40,41,42,43,44]. GaS is an indirect bandgap semiconductor which possesses a crystal lattice with a hexagonal structure, each layer consisting of two Ga and two S closed-packed sublayers in the stacking sequence of S–Ga–Ga–S along the c axis [45,46,47]. Recent experimental progress has achieved the preparation of two-dimensional GaS sheets, revealing its excellent characteristics, and opening up huge opportunities for the possible applications of photodetectors, field-effect transistors, and electroluminescent devices [48,49]. Additionally, it exhibits high optical response and photoresponsivity over a wide spectral range [50]. The phonon properties and temperature dependence of GaS thin films have recently been demonstrated, revealing the influence of substrate and the number of layers on the performance of GaS sheets [51]. Because of its graphene-like hexagonal structure, it can be predicted that there are still more application prospects to be explored [52,53]. Different atomic structures of GaS can be obtained by different growth methods [54,55,56]. Considering the application potential of GaS in the field of nonlinear optics, there is a strong motivation for one to improve the quality of GaS in terms of size and purity [57].

Here, high-quality GaS was synthesized via the chemical vapor transport method. The fabricated GaS crystal has a layered structure with a weak interlayer coupling of vdW force, which is easy to be cleaved to synthesize nanosheets. The integration of GaS-SA and the optical fiber is realized by transferring the sheet material to the end cap of optical fiber through Polydimethylsiloxane (PDMS) auxiliary technology. A GaS-SA based mode-locking laser with different preparation processes and integration methods than reported works at telecommunication band shows the rich generation ways and better application prospects of GaS-based devices. The soliton mode-locked laser was realized with a central wavelength of 1561 nm at a repetition rate of 16.6 MHz. The pulse duration of 448 fs is achieved with a maximum energy of 0.48 nJ, yielding a peak power of 1.08 W. The long-term stability and the reliability of the GaS-SA for mode-locking operation are confirmed by monitoring the output performance of the laser over 30 days. Experimental results may stimulate one to explore the GaS-SA in multi-function ultrafast high-power laser applications.

## 2. Materials and Methods

### 2.1. Crystal Growth

The GaS single crystal was grown via the chemical vapor transport technique. The technique is as follows: First, about 0.5 g of the elements (Ga: 99.999% and S: 99.999%) were introduced into a quartz tube (20 mm in diameter, 16 cm in length). Then evacuated to a pressure of ~10^−6^ Torr to avoid explosions due to the strong reaction and sealed. The mixed powder reacts at 900 °C for 7 days. After cooling down to room temperature, hexagonal transparent yellow flakes could be obtained. The fabricated GaS crystal has a layered structure with a weak interlayer coupling of vdW force, which is easy to be cleaved to synthesize nanosheets.

### 2.2. Apparatus and Characterization

**Figure 1:**
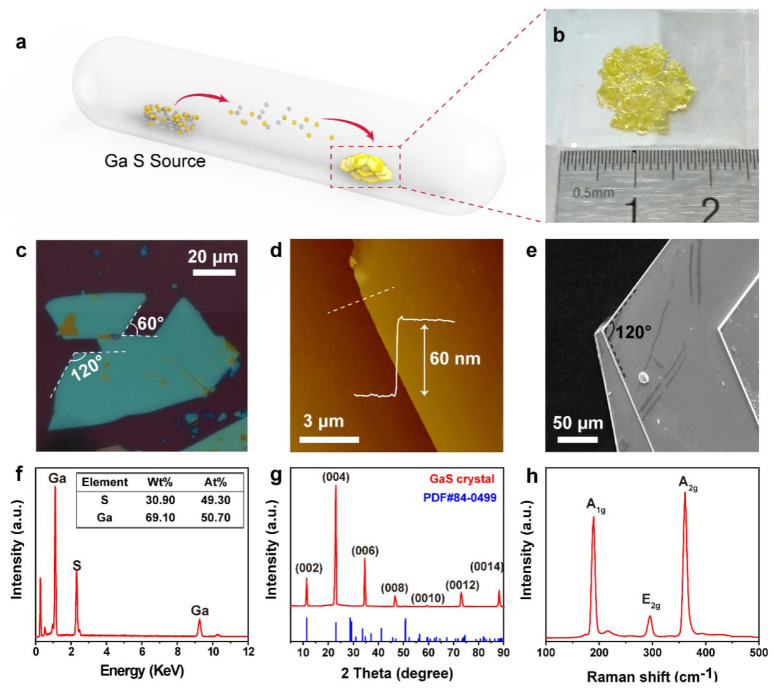
High-quality hexagonal structured GaS crystals synthesis and characteristics. (**a**) Schematic diagram of GaS crystal growth via a chemical-vapor transport (CVT) method. (**b**) Image of GaS flake next to the steel ruler. (**c**) Mechanically stripped GaS crystals under microscope. (**d**) AFM height profile of the GaS nanosheet on a SiO_2_/Si substrate. (**e**) Secondary electron image. (**f**) The EDX spectra. (**g**) X-ray diffraction pattern of GaS crystals. (**h**) Raman spectra of few-layer GaS nanosheets.

Figure 1a shows the schematic image of GaS crystal growth process. During the whole process, the raw materials placed at the high-temperature side will transport to the low-temperature end under the temperature gradient. The iodine (I_2_) was adopted as a transport agent. After that, GaS crystal was obtained without any further wash in solvent for the removal of residual impurities. Figure 1b shows a photograph of the GaS crystals next to the steel ruler. Here, the GaS crystals present a transparent yellow luster and flake shape. The bulk size is over 10 mm, indicating a high crystal yield. As shown in Figure 1c, the GaS material is easy to obtain large-size films (~200 um on the SiO_2_/Si substrate) by mechanical stripping method [58], and most of them hold a corner of 60°. An atomic force microscope (AFM, Dimension 3100, Bruker, Billerica, MA, USA) was utilized to test the sample thickness. The representative height profile of an exfoliated GaS nanosheet is presented in Figure 1d, the measured thickness is ~60 nm, also showing a uniformly clean surface. Scanning electron microscopy (SEM, S4800, HITACHI, Tokyo, Japan) was carried out to characterize the morphology and chemical compositions of GaS. The surface topography is uniform, revealed by a secondary electron image (Figure 1e). The GaS flake exhibit a hexagonal shape and typical lamellar structure. The energy dispersive X-ray (EDX, S4800, HITACHI, Tokyo, Japan) spectra shown in Figure 1f represented the material with atomic ratios of 49.3% (Ga), 50.7% (S), which is in agreement with the stoichiometric ratio of 1:1. The X-ray diffraction (XRD, AXS D8 Advance, Bruker, Billerica, MA, USA) measurement was further conducted by an X-ray scattering system with Ni-filtered Cu K radiation to study the crystal structure. Figure 1g displays the characteristic peaks of prepared GaS nanoflakes. Unlike powder diffraction, the characteristic peaks dominantly directed to the crystal planes along the c-axis. These obvious and sharp (00*l*) diffraction peaks correspond to the standard PDF card for GaS (PDF #84-0499), indicating the sample is in high crystallinity. Raman spectra were obtained via a LABRAMs HR Evolution instrument, HORIBA, France, and the visible laser (*λ* = 532 nm) was used as a probe. There are three Raman active modes, one in-plane mode (E_g_), two out-of-plane modes (A_g_), as reported in other work [59]. As Figure 1h illustrates, the A_1g_ (187.5 cm^−1^), A_2g_ (374.8 cm^−1^), and E_2g_ (303.75 cm^−1^) vibrational peaks. All of these data prove that the growth parameter settings in this experiment assure the controllable preparation of high-quality GaS crystal.

**Figure 2:**
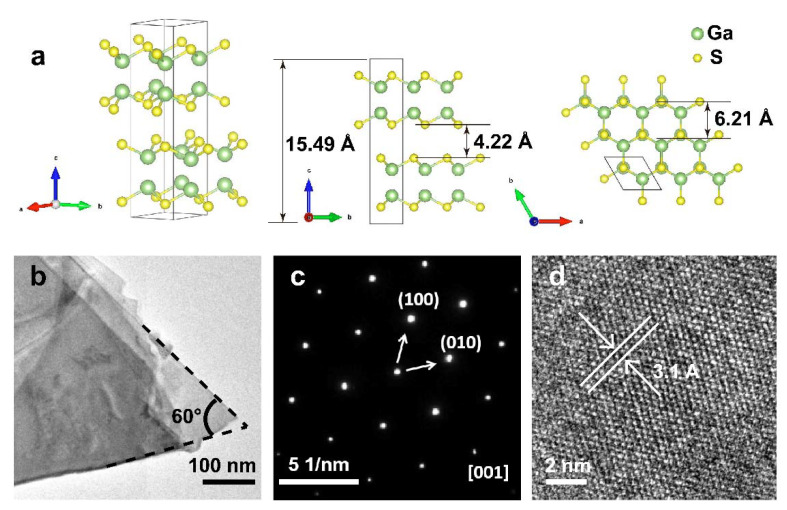
Crystals structural of the layered GaS crystals. (**a**) schematic of the atomic structure of GaS (left: middle: side-view, right: top-view). The big green ball represents Ga atom, and the small yellow ball stands for S atom. (**b**) Low-magnification TEM image. (**c**) SAED pattern. (**d**) High-resolution TEM image.

To further study the microscopy arrangement of the GaS crystal, the layered GaS bulk were deformed in isopropanol solvent. Then, the nanosheets were transferred onto the copper mesh and characterized by a transmission electron microscope (TEM, Tecnai G2 F20 S-Twin, FEI, Hillsboro, OR, USA). Figure 2a presents the theoretical lattice structure model, which shows the arrangement of atoms from different perspectives. Representatively, the GaS has a hexagonal crystal structure and belongs to the space group of P63/mmc. In a structure unit, the lattice constant a = b = 3.58 Å, c = 15.49 Å. c has about two fundamental layers in thickness. Within GaS layers, S atoms and Ga atoms are covalently bonded, forming a honeycomb structure. The individual GaS layers are connected by weak van der Waals interactions. Figure 2b is the morphology of a GaS nanosheet at a low magnification, and its thickness is relatively thin, resulting in higher transparency. The selected area electron diffraction (SAED, Tecnai G2 F20 S-Twin, FEI, Hillsboro, OR, USA) pattern showed sharp and well-arranged diffraction spots in a hexagonal pattern (Figure 2c), suggesting the high crystalline quality of the as-prepared nanosheet, which matches well with the planes in the XRD data. Moreover, the high-resolution TEM (HRTEM) image of a representative GaS nanosheet is shown in Figure 2d; it matches with the schematic model in Figure 2a (the top view). The lattice fringes marked here has a spacing of 3.1 Å, it can be indexed to the crystal planes of (100) or (010) along the zone axis of [001].

**Figure 3:**
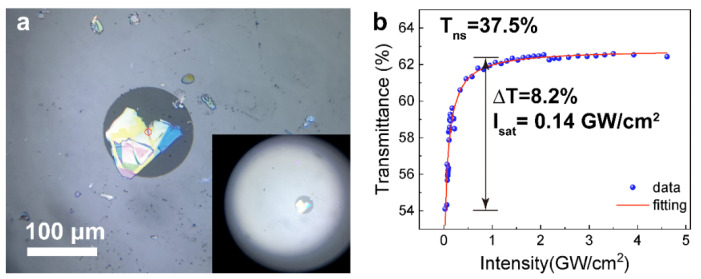
Characteristic of the GaS-SAs. (**a**) The optical microscope digital image of GaS nanosheets attached on the fiber end face. Inset: The panorama of the surface of the optical fiber connector. The bright spots near the center are GaS nanosheets. (**b**) Nonlinear transmission of the GaS-SA.

The integration of SA and the optical fiber is realized by transferring the nanosheet material to the end cap of optical fiber through Polydimethylsiloxane (PDMS) auxiliary technology. AFM characterization with different colors was carried out to establish a thickness reference (Supplementary Materials, Figures S1 and S2). As can be seen from the camera images shown in Figure 3a, a piece of GaS nanosheets is attached to the fiber end surface, the thickness of the nanosheet that covers the core is ~40 nm (about 52 layers after calculation). The outer diameter of the cladding is ~125 μm and the core diameter is ~9 μm. A red circle aims to show the location of the core which shows that the fiber core has been completely covered by GaS nanosheets. The properties of the corresponding saturable absorbers in the 1.55 μm wavelength region were tested by a double arm detection technique [27]. The transmittance of an ideal saturable absorber can be described as follows:

$$T\left(I\right)=1-\Delta T\times \exp(-\frac{I}{I_{sat}})-T_{ns}$$

where *T(I)* corresponds to the intensity-dependent transmittance, Δ*T* is the modulation depth, *I* denotes the incident light intensity, *I_sat_* is the saturation intensity of saturable absorber, and *T_ns_* is the non-saturation loss.

The measured nonlinear transmittance under different incident power intensities is presented in Figure 3b and the transmission curve is fitted with a typical SA function, revealing that the GaS-SA has a modulation depth (Δ*T*) of 8.2% and a saturated intensity (*I_sat_*) of 0.14 GW/cm^2^, respectively. Linear and nonlinear measurements of GaS with other thickness is shown in Supplementary Materials, Figures S3 and S4.

### 2.3. Density Functional Theory Analysis and Saturable Absorption Mechanism

**Figure 4:**
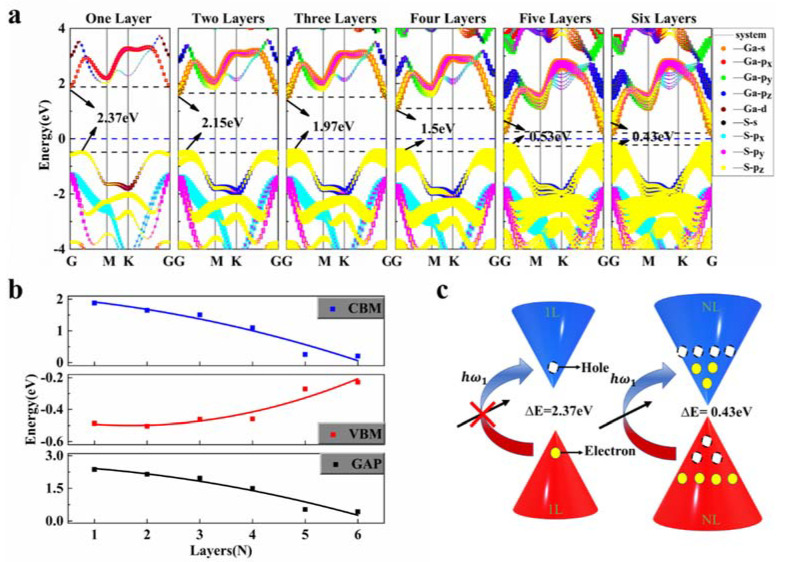
The PBE calculated band structure (**a**) 1–6 Layers GaS, the size of the dots in the bands indicates the weight of the electronic orbital. (**b**) The dependence of band gap, VBM and CBM on number of layers. (**c**) Schematic diagram of photogenerated electron leap in monolayer and multilayer GaS.

In order to investigate the saturable absorption mechanism, the Perdew-Burke-Ernzerhof (PBE) exchange-correlation function is used in coupled with density generalized functional theory (DFT) methods to perform the calculations. The light absorption process of a material is closely coupled with its electronic structure, and Figure 4a shows the electronic band structure of GaS under different layers. It can be found that GaS is an indirect bandgap semiconductor, which is consistent with previous work. Its conduction band minimum (CBM), dominated by the s orbit of Ga and the p_z_ orbit of S, is at the G point, while its valence band maximum (VBM) is between the paths G-M, mainly contributed by the p_z_ orbit of S. According to the selection rule for the electromagnetic transitions (that is, the electron absorbing photons of VBM ($S-|p_{z}\rangle$) are more likely to leap to CBM (Ga−|s〉), this indicates that even if GaS possesses an indirect band gap, photoelectrons can make effective jumps, making GaS have good light absorption ability. On the other hand, it can be seen from Figure 4b that the band gap of GaS has a strong dependence on the number of layers. Due to interlayer coupling, the band gap varies dramatically from 2.37 eV in single-layer structures to 0.43 eV in multi-layers, which is especially strong for the modulation of CBM. And as the number of layers increases, the number of energy bands also increases, and the number of electronic states that can be occupied also increases, so the absorption of light is further enhanced, as shown in the schematic diagram in Figure 4c. This illustrates that the GaS material can saturate the laser at a 1.55 μm wave band regime.

## 3. Experimental Setup and Results

**Figure 5:**
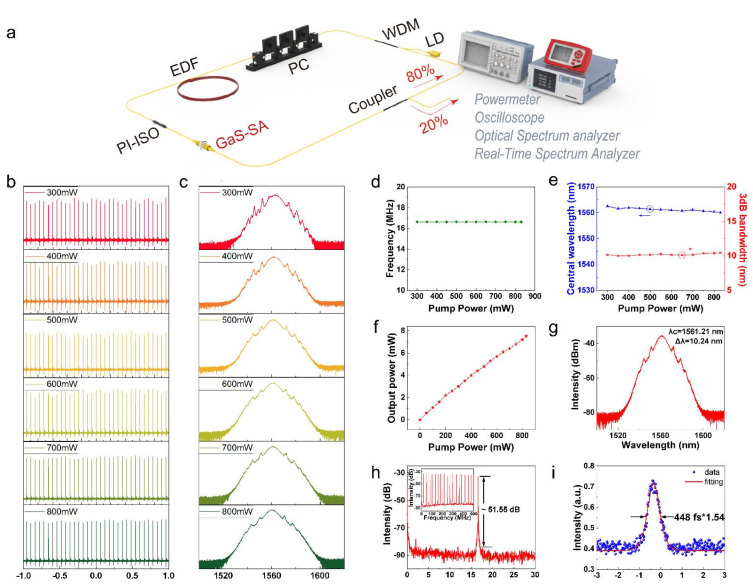
Characteristic of the femtosecond pulse generated from the Er-doped mode-locked fiber laser based on the GaS-SA. (**a**) Configuration of the laser ring cavity oscillator. (**b**,**c**) Pulse train and spectrum under different pump power. (**d**,**e**) Pulse repetition rate, central wavelength and 3 dB bandwidth as a function of pump power. (**f**) Output power vs. pump power. (**g**) Typical mode-locking optical spectrum at 550 mW pump power. (**h**) The corresponding radio frequency (RF) spectrum at f = 16.67 MHz. Insert: RF spectrum within 500 MHz range. (**i**) Typical autocorrelation trajectory of emission pulse at 830 mW.

Figure 5a depicts the schematic of the GaS-based passively mode-locked fiber laser in which a piece of 2.2 m EDF serves as the gain medium. The cavity is pumped by a 976 nm laser diode (LD) with a maximum power of 830 mW through a 980/1550 nm wavelength-division multiplexer (WDM) which is followed by a polarization controller (PC), which was utilized to manipulate the polarization state in cavity. A polarization-independent isolator (PI-ISO) placed after the EDF ensures the unidirectional light propagation in the cavity. As a result, an 80% portion of the pump light was absorbed by the EDF and then passed through the GaS-SA, reducing the risk of damage to SA devices. A 20:80 optical coupler extracts 20% of the intra-cavity energy from the laser cavity for measurements. The pigtails of all the elements are single-mode fiber (SMF) and the cavity length is 12 m. The dispersion coefficient of SMF-28 and EDF is −22.94 ps^2^/km and 28 ps^2^/km, respectively. The net dispersion in the cavity is the sum of the dispersion of the respective fibers. Hence the total cavity dispersion is calculated to be −0.163 ps^2^.

Stable mode-locked ultrafast pulse appeared in the GaS-SA based fiber laser when pump power was increased to 300 mW at an appropriate polarization state. The mode-locked pulse train is presented on the top panel in Figure 5b and the corresponding spectrum is shown in the top panel in Figure 5c. The pulse train measured by oscilloscope is with an interval of approximately 60 ns, corresponding to a fundamental repetition rate of 16.67 MHz, which is determined by the physical length of the laser ring cavity. The spectrum is broader with clear Kelly sidebands sitting on both sides of the spectrum, confirming the anomalous dispersion regime in the cavity. Remarkably, further increasing the pump power up to 800 mW doesn’t destroy the mode-locking operation (See Figure 5b,c). Under a lower pump power, there is an obvious amplitude jitter in the time domain featuring an appearance by secondary sidebands entrained by the Kelly sidebands in the spectrum, whereas at high pump power the amplitude jitter of the pulse train is significantly reduced, which leads to a smooth spectrum with clear symmetrical Kelly sidebands in the spectral domain, demonstrating the stable mode-locking operation at a high pump power.

It is worth noting that the GaS-SA enables the laser to operate in the fundamental mode-locking regime at high power, as evidenced by the recorded constant pulse repetition rate of 16.67 MHz at a different pump power shown in Figure 5d. In the process of the increase in the pump power from 300 mW to 830 mW, the central wavelength almost remains unchanged (See blue curve in Figure 5e). In the meantime, Figure 5e depicts the 3 dB bandwidth changes little but shows a potential growth trend (maximum range 0.42 nm). Figure 5f shows that the output power increases linearly in accordance with increasing the pump power. The maximum output power is ~8 mW in our experiments, corresponding to a pulse energy of 0.48 nJ and peak power of 1.08 W.

**Figure 6:**
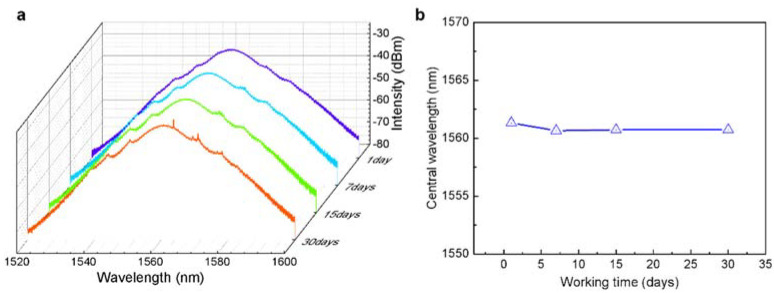
Long-time monitor of the GaS-based mode-locking pulse fiber laser. The performance of the (**a**) spectrum and (**b**) central wavelength after 1, 7, 15, 30 days.

A typical spectrum in the mode-locking regime at a pump power of 550 mW is elucidated in Figure 5g, exhibiting a set of clear Kelly sidebands that evidences the stable mode-locking operation. The corresponding radio frequency (RF) spectrum recorded in a range of 30 MHz with the resolution bandwidth (RBW) of 50 Hz exhibits a single peak at 16.67 MHz with a high signal-to-ration (SNR) of 51.55 dB, which proofs the single pulse operation. The inset in Figure 5h is the RF spectrum within the 500 MHz ranges also confirms the stability of the mode-locking operation. Figure 5i shows the pulse autocorrelation (AC) trace measured by an autocorrelation instrument. The pulse duration is with the full width at half maxima (FWHM) of 448 fs. The coefficient 1.54 is the conversion coefficient of pulse duration between the actual pulse width and the displayed pulse width of autocorrelation trace [60]. When pump power is 830 mW, the time-bandwidth product (TBP) is 0.576, compared to the TBP of the hyperbolic secant pulse being about 0.315, which indicates that there is a chirp in the pulse and the pulse width has room for continuing compression. The long-term performances of the laser are characterized by monitoring the output from the laser cavity over 30 days and the experimental observation confirms the long-term stability and reliability of such a GaS-SA for mode-locking (See Figure 6).

The overall performance of our GaS-SA-based Er-doped mode-locked fiber laser is summarized in the below table and is compared to the performances of different two-dimensional materials-based mode-locked lasers reported in the literature. As shown in Table 1, obviously, a shorter pulse width and equal modulation ability are obtained in the Group III metal chalcogenides. Additionally, our work obtained a higher SNR RF and robust stability in the GaS-based EDF laser, compared with the same group of compounds. These results trigger the discovery of optical properties and the nonlinear optical absorption of Group III metal chalcogenides, and shows further potential for the research of pulsed lasers based on nanomaterials.

## 4. Conclusions

In summary, we successfully synthesized high-quality hexagonal structured GaS crystals using a CVT method. Hence, large area thin-film GaS nanosheets were readily prepared using a direct exfoliation method. We have fully characterized the optical properties of GaS-SA and nonlinear optical absorption, which demonstrates the potential of ultrafast pulse generation. A GaS-SA element has been inserted in an Er-doped all-fiber laser for the generation of ultrashort pulses. Femtosecond (448 fs) soliton pulse output with long-term (30 days) stability was obtained in the Er-doped fiber laser. We note that the performance of the GaS-SA-based mode-locked lasers can be improved by various approaches, for instance using a dispersion management or nonlinearity management technique. Hence, the experimental results not only show the great potential of GaS-SA for ultrashort pulse generation at 1550 nm but also encourages the exploration of types of SAs in other wavelength ranges, for instance in the 2 μm or MID-IR. Furthermore, our experimental results may promote the optical community’s interest in the Group III metal chalcogenides family for nonlinear optics.

## Figures and Tables

**Table 1:** Performance comparison of fiber lasers based on different low-dimensional materials.

SA		*I_sat_* [MW/cm^−2^]	Δ*T* [%]	*λ* [nm]	*t_min_* [fs]	*F* [MHz]	SNR [dB]	*E_max_* [nJ]	Reported Work Duration	Ref.
Gra	Graphene	0.61/0.71	6.2	1565	756	1.79	65	\	\	[16]
BP	Black phosphorus	8.23	9.98	1567	538	30.3	60	\	200 h	[61]
TIs	Bi_2_Se_3_	12	3.9	1557.5	660	12.5	55	0.144	8 h	[62]
TMDC	VSe_2_	61.9	22.5	1565.69	910	2.081	76	\	24 h	[63]
MXene	Ti_3_C_2_T_x_	1.94	11.3	1564	597	18	55.2	\	\	[64]
other	Cr_2_Ge_2_Te_6_	5.8	15.3	1561.59	881	19.33	48	0.149	\	[65]
Group III metal chalcogenides	GaSe	0.024	10	1501–1586	3.6 µs	0.058	47	30	Q-switched	[66]
	GaTe	3100	1.27	1530.9	115	8.79	43	0.436	9 h	[67]
	InSe	\	9.55	1568.73	2.06 ps	1.73	\	\	\	[68]
	TIS_2_	17.79	13.19	1531.69	2.36 ps	3.43	60	0.05	\	[43]
	GaS	140	8.2	1560	448	16.6	51.55	0.48	30 days	Our work

Note: *I_sat_* = saturable intensity; Δ*T* = modulation depth; *λ* = central wavelength; *t_min_* = minimum pulse duration; *F*= repetition rate; *E_max_* = maximum pulse energy.

## Data Availability

Not applicable.

## Supplementary Materials

The following supporting information can be downloaded at: https://www.mdpi.com/article/10.3390/nano12030378/s1, Figure S1: Thickness comparison color card of GaS nanosheets; Figure S2: Optical images transferred from GaS with different thickness to the end face of fiber; Figure S3: Linear optical absorption spectrum of the GaS nanosheets with different thickness; Figure S4: Nonlinear absorption properties of the fiber integrated GaS nanosheets.
