# Supplementary material for: Synthesis of Hexagonal Structured GaS Nanosheets for Robust Femtosecond Pulse Generation
Source: Nanomaterials (Basel). 2022 Jan 24;12(3):378. doi: 10.3390/nano12030378 (PMC8839219; doi:10.3390/nano12030378)
Supplement: Supplementary file 1 [file nanomaterials-12-00378-s001.zip › nanomaterials-1498398-supplementary.pdf]

nanomaterials

Supplementary Materials

Synthesis of Hexagonal Structured GaS Nanosheets for Robust Femtosecond Pulse Generation

Kun Guo [1], Qiang Yu [1], Fangqi Liu [2], Haiqin Deng [1], Tianan Yi [3], Bo Ren [1], Wei Su [3], Sicong Zhu [2], Zhiqiang Wang [4,5,*], Jian Wu [1,*] and Pu Zhou [1]

[1] College of Advanced Interdisciplinary Studies, National University of Defense Technology, Changsha 410073, China; guokun16@163.com (K.G.); qyu2015@sinano.ac.cn (Q.Y.); ddhq9859@163.com (H.D.); rbone725@163.com (B.R.); zhoupu203@163.com (P.Z.)
[2] Hubei Province Key Laboratory of Systems Science in Metallurgical Process, College of Science, The State Key Laboratory for Refractories and Metallurgy, Wuhan University of Science and Technology, Wuhan 430081, China; lfq7@wust.edu.cn (F.L.); sczhu@wust.edu.cn (S.Z.)
[3] College of Mechanical and Electrical Engineering, Hohai University, Changzhou 213022, China;
[4] Aston Institute of Photonic Technologies, Aston University, Birmingham B4 7ET, UK
[5] Advanced Photonic Technology Lab, College of Electronic and Optical Engineering, Nanjing University of Posts and Telecommunications, Nanjing 210023, China
* Correspondence: z.wang35@aston.ac.uk (Z.W.); wujian15203@163.com, wujian15@nudt.edu.cn(J.W.)

Citation: Guo, K.; Yu, Q.; Liu, F.; Deng, H.; Yi, T.; Ren, B.; Su, W.; Zhu, S.; Wang, Z.; Wu, J.; et al. Synthesis of Hexagonal Structured GaS Nanosheets for Robust Femtosecond Pulse Generation. *Nanomaterials* **2022**, *12*, 378. https://doi.org/10.3390/nano12030378

Academic Editors: Werner Blau and Bouchta Sahraoui

Received: 22 November 2021
Accepted: 21 January 2022
Published: 24 January 2022

Publisher's Note: MDPI stays neutral with regard to jurisdictional claims in published maps and institutional affiliations.

Copyright: © 2022 by the authors. Licensee MDPI, Basel, Switzerland. This article is an open access article distributed under the terms and conditions of the Creative Commons Attribution (CC BY) license (https://creativecommons.org/licenses/by/4.0/).

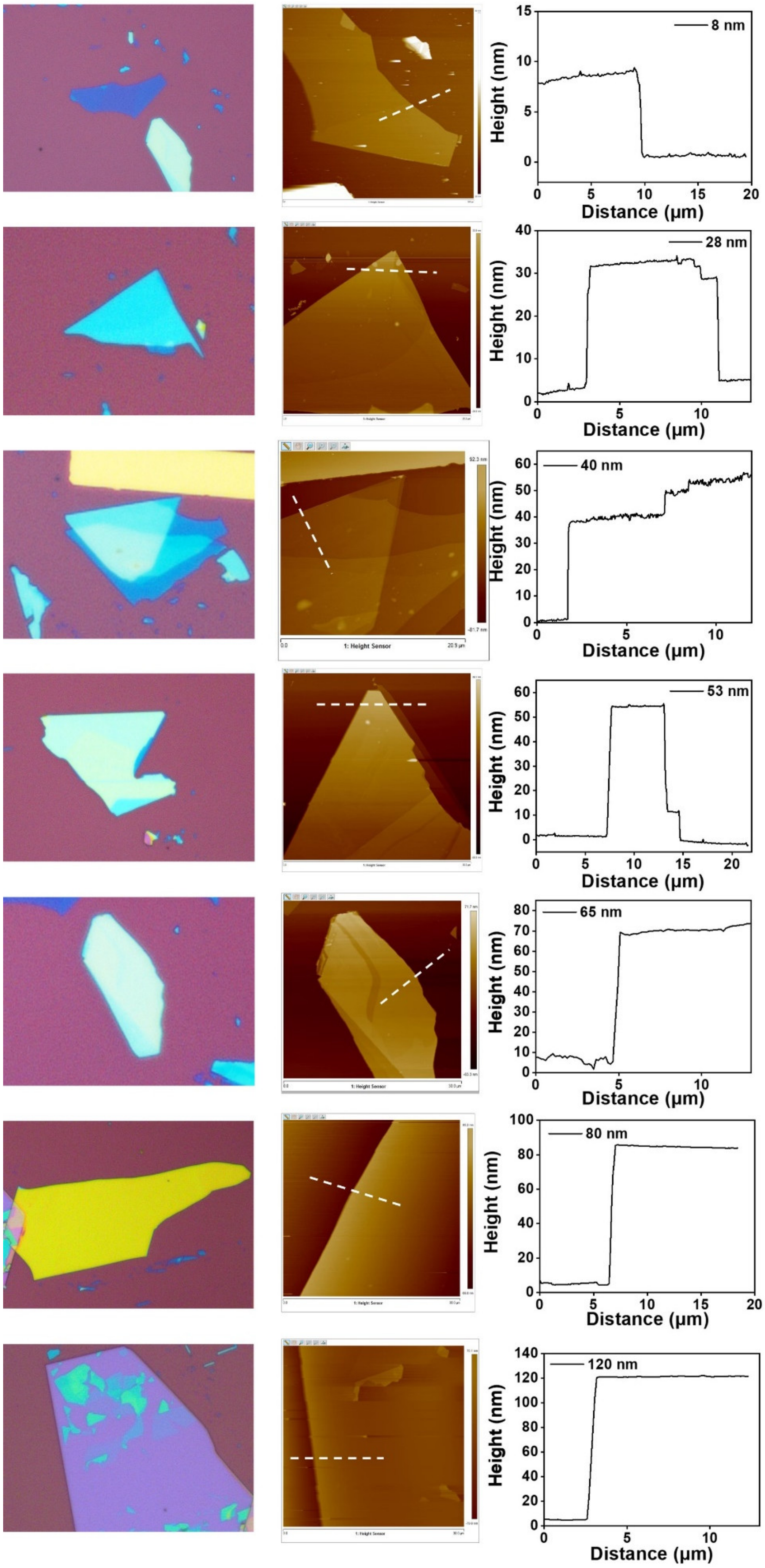

Figure S1. Thickness comparison color card of GaS nanosheets.

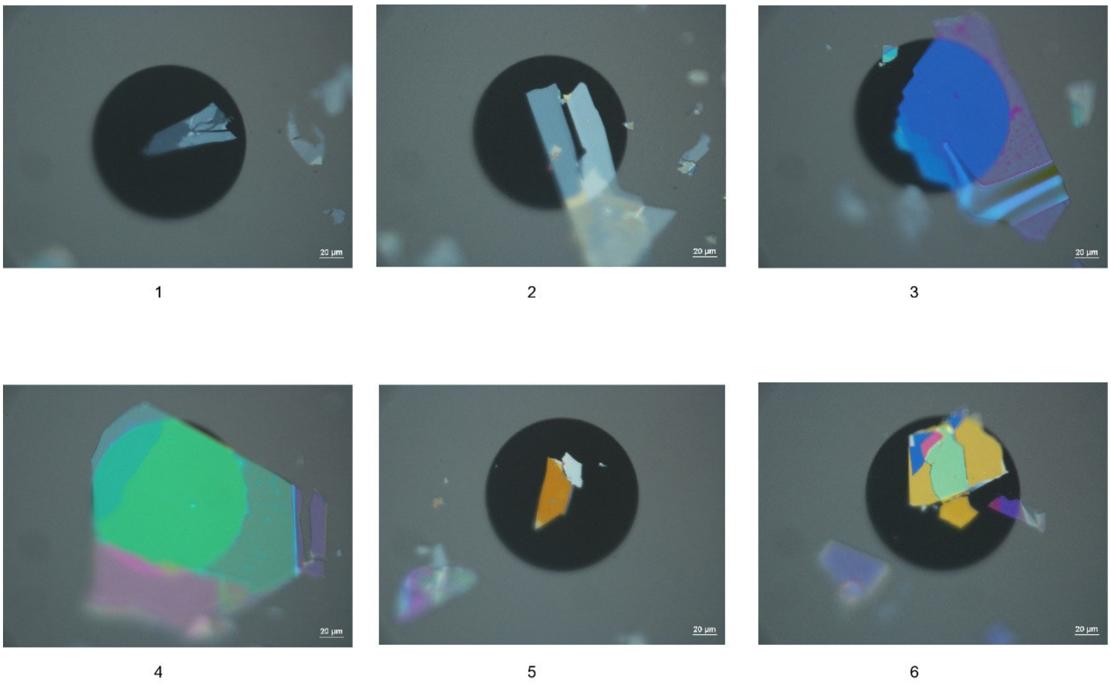

Figure S2. Optical images transferred from GaS with different thickness to the end face of fiber.

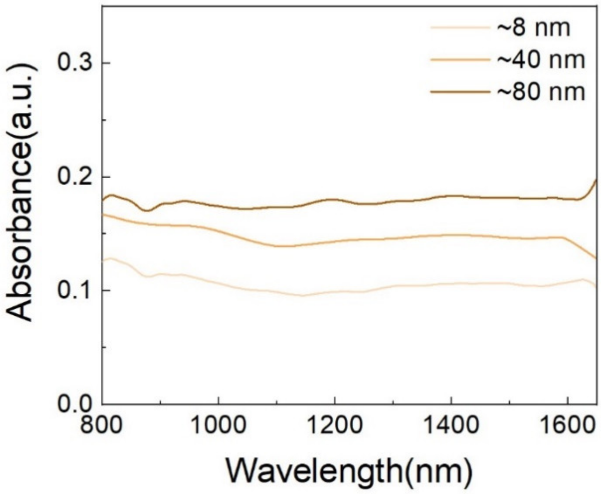

Figure S3. Linear optical absorption spectrum of the GaS nanosheets with different thickness.

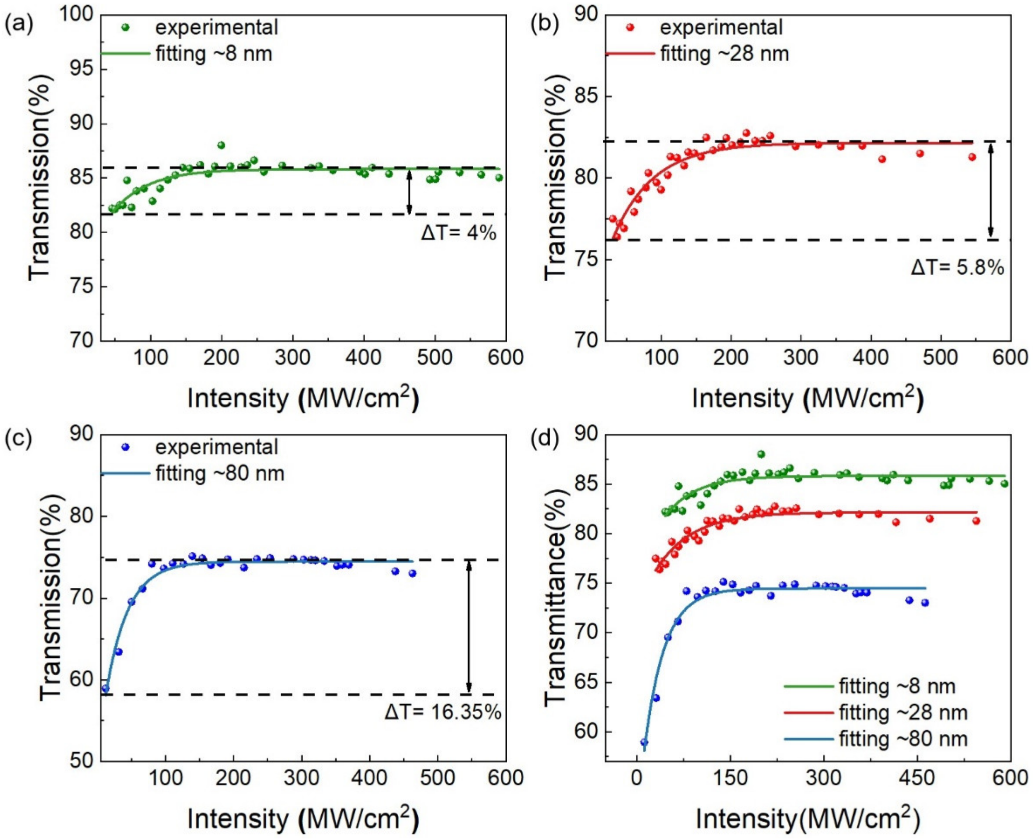

Figure S4. Nonlinear absorption properties of the fiber integrated GaS nanosheets. (a–c) Transmission as a function of the incident intensity for different thicknesses under 1560 nm laser excitation. (d) Contrast curve.
